# The Responsibility of the Insane
*1. Medical Testimony and Evidence in Cases of Lunacy. By Thomas Mayo, M.D., F.R.S. London: John W. Parker and Son, 1854.2. Unsoundness of Mind in relation to Criminal Acts; being the *Sugden Prize Essay* for 1854. By John Charles Bucknill, M.D., Physician to the Devon County Lunatic Asylum. London: Samuel Highley, 1854.3. Unsoundness of Mind in its Medical and Legal Considerations. By J. W. Williams, M.D. *Dublin Quarterly Journal of Medical Science*, Nos. XXXV., XXXVI., XXXVII. 1854-5.


**Published:** 1855-04-01

**Authors:** 


### Art. II.—THE RESPONSIBILITY OF THE INSANE.*

What is the condition that releases a man from responsibility to the laws of society?

Notwithstanding many conflicting arguments and decisions, medical and legal, we may affirm that this question is practically solved, both according to the practice of the law and the dictates of common justice, when insanity is established. Although not convertible terms, insanity and irresponsibility are inseparably associated. Irresponsibility follows upon insanity as a logical necessity. We should, therefore, hold that the difficulty would be overcome could we expound clear and definite rules by which insanity might be known. But, in discussing this question, so momentous to society and to individuals, with the authors the titles of whose works are quoted below, we shall be compelled to consider it under another and still more complicated aspect. Dr. Mayo and Dr. Bucknill, in express terms, contend that there are certain states in which responsibility is only modified, and not annulled. They would break down the old barrier which has hitherto been deemed the natural, and served as a recognizable boundary between responsibility and irresponsibility. That barrier removed, we shall have to call upon Dr. Mayo and Dr. Bucknill to show us how far we are to travel along the road beyond the confines where sanity ends and insanity begins before we arrive at the vanishing point of responsibility. We shall have to ask them to define at what degree of madness they would interfere to stay the infliction of punishment. We greatly fear that, the only intelligible landmark removed,

* 1. Medical Testimony and Evidence in Cases of Lunacy. By Thomas Mayo, M.D., F.R.S. London: John W. Parker and Son, 1854.

2. Unsoundness of Mind in relation to Criminal Acts; being the *Sugden Prize Essay* for 1854. By John Charles Bucknill, M.D., Physician to the Devon County Lunatic Asylum. London: Samuel Highley, 1854.

3. Unsoundness of Mind in its Medical and Legal Considerations. By J. W. Williams, M.D. *Dublin Quarterly Journal of Medical Science*, Nos. XXXV., XXXVI., XXXVII. 1854-5.

we shall, like benighted travellers, have to grope our way in a dreary wilderness of speculation,—no point of departure, no guiding star, no compass, no resting-place, no progress, and no goal! Can the search for the conditions of responsibility be hopeful or profitable if thus undertaken? We fear not; but, nevertheless, we cannot shrink from the task of examining the proposition as presented to us by the authors we have named. Nor do we desire to shrink from it. The doctrine maintained by them is fraught with consequences to society too mighty, and, we think, perilous, to permit us, in conscience or in inclination, to evade it, or to pass it by without ample investigation.

We would not call down upon these authors the penalties invoked by the ancients against those who removed the Terminal Gods. We venerate the principle of liberty of thought; but we cannot, reflecting upon the subversive tendency of the doctrine we are about to discuss, avoid uttering our deep conviction that it is one that ought not to be promulgated unless supported by very cogent arguments.

In entering upon the task as presented to us, we are still, however, not freed from the necessity of seeking to determine the essential conditions of insanity as the first step in the inquiry. Even the authors we have named, although contending that responsibility passes into the cloudy regions of insanity, and unable, as may well be imagined, to indicate at what precise step of insanity responsibility ends, cannot escape from this elementary necessity. We could not, indeed, appreciate their reasons, or do justice to their arguments, unless we began by inquiring what it is that they understand by the term, insanity.

Both have, with a fatal courage, dared to enunciate definitions. Every man who throws down a definition challenges criticism. But first, we think it our duty to cite the passages in which they explain their doctrine of responsible madness. Dr. Mayo is precise, and even dogmatic. There is none of the diffidence of doubt; he lays down his doctrine with all the assurance of a man who has established it on the basis of scientific demonstration, with all the confidence of a Columbus who has made a discovery in advance of the intelligence of the age in which he lives. In his preface, Dr. Mayo tells us—" In the course of my inquiries I have been led to certain conclusions, for which I cannot expect a cordial or immediate reception." "The second (of these conclusions) arises out of the question whether some offences of the insane ought not to be visited with some form of secondary punishment." "The law will remain a dead letter, or be continually ignored by the sympathies of judges, juries, and, I may add, of medical witnesses, unless some practical distinction can be arranged which may enable the responsible insane to undergo some lower degree of punishment than that inflicted on similar delinquents being of sound mind,"

(p. 51.) Dr. Bucknill says—"It is the system of the English law to allow no degrees of responsibility. A criminal is either responsible, or he is irresponsible. . . . We are extremely happy to observe that in Ireland the administration of the law, practised with such inflexibility in England, is occasionally departed from; and, in such cases as those of William Quinlaw, L. Grady, and others, mentioned in the Inspector's reports, the Lord Lieutenant has sometimes commuted the sentence of death into transportation, on the ground of *imperfect* responsibility." He proceeds to quote, with approbation, the following extraordinary doctrine of Drs. White and Nugent:—"If there are extenuating circumstances connected with the psychological condition" (meaning, if they are insane) "of the accused, they are legitimate subjects, to be considered in meting out the after-punishment, but certainly not, in the first instance, for an unqualified acquittal." Hence Drs. White and Nugent—and Dr. Bucknill approves—see no inconsistency, nothing revolting to common sense or justice in putting an admitted lunatic upon a solemn trial for his life. Both Dr. Mayo and Dr. Bucknill lay some stress upon the French custom of making gradations of punishment by admitting "extenuating circumstances," and seek to force a kind of analogical argument in favour of their position out of this practice. But is it not a palpable *non sequitur* to apply a rule, that is not without reason when sane people are concerned, to cases of undoubted insanity? It is surely a singular solecism in jurisprudence to admit insanity as an "*extenuating* circumstance." The instance of Henriette Cornier, cited by Dr. Mayo, is not, as he supposes, a proof that insanity has been admitted by the French law as an extenuating circumstance in the light of a principle of law, but simply one of those compromises between the uninformed judgment of juries whose insufficient faith in the decision of medical science is sometimes unable to credit testimony as to the existence of insanity, and their natural sense of justice, which forbids them to visit the full penalties for crime on the heads of wretches whose responsibility is a matter of dispute.

But there is one passage in Dr. Williams' essay—an essay which for sound and original argument we cannot too highly commend—which would seem to imply that he also is disposed to admit a limited responsibility as attaching to the insane: "Where does the ability to determine on a particular act cease or commence? For accordingly must be the responsibility or irresponsibility of the offender. Experience and observation prove that this mental regulation is, to a certain extent, under a man's own control; it therefore follows that those illegal acts resulting from unsoundness of mind within the individual regulations of the will are justly regarded as crimes, while similar acts, originating from mental conditions in which the will has no part, cannot be esteemed as other than so many evidences of insanity." (*Dublin Quarterly Journal*, p. 267, Nov. 1854.) But this seems to be put theoretically rather than as a practical law, and is not carried out by the general argument of Dr. Williams' excellent treatise.

The doctrine of responsible insanity, then, as upheld by Dr. Mayo and Dr. Bucknill, is now before our readers. We proceed to state their definitions of insanity; and then we shall endeavour to trace, as well as we can, what kinds and degrees of insanity, according to these authors, involve responsibility.

Dr. Mayo thus explains his views :—"Now, in looking for a term which may contain the essential mental elements of insanity, and, therefore, contain a criterion of its presence, I adopt delirium, as used by M. Pinel and Dr. Cullen" (p. 13). "The second phase of delirium consists in the presence of certain delusions, or false perceptions, of which there are two principal forms." The first, in which the delusion may simulate a perception of the special senses, is called by Dr. Mayo "objective delusion." This is the hallucination of French authors. Secondly, "the delusion or false perception may have no direct reference to objects of sense, but may, apparently, turn on perceptions of the understanding alone, thus embracing a large, and, I regret to say, indefinite category, which contains preposterous notions respecting power, station, conduct, moral motives, future prospects," &c. Delusions of this nature Dr. Mayo calls "notional,"—a not very felicitous term.

We postpone, for the present, criticism upon this position, and place by the side of it the definition of Dr. Bucknill. According to this acute and experienced psychopathist disease is a necessary condition of insanity. "A change, therefore, with impairment or perturbation of function, is the chief test of centro-mental disease," (p. 33.) "Insanity may be intellectual, emotional, or volitional, and though in the concrete it is not easy to find pure and unmixed cases under either of these heads, such cases do occasionally subject themselves to observation. . . . Insanity, therefore, may be defined as a condition of the mind in which a false action of conception or judgment, a defective power of the will, or an uncontrollable violence of the emotions or instincts have, separately or conjointly, been produced by disease," (p. 28.) We do not consider it important to adduce Dr. Bucknill's definition of delusion, since, according to the above definition of insanity, delusion *per se* does not imply insanity: disease must be coexistent. But since disease can, in many cases, only be presumed to exist on the evidence of intellectual alienation, we think it useful to quote the characteristic features of insane delusions as laid down by Dr. Bucknill. "1st. The delusions of the insane are generally independent of the opinions of others; they isolate the person who entertains them from his kind: whereas the sane portion of mankind are gregarious in their absurdities. . . . 2nd. The faith of the insane in their delusive opinions is stedfast and unflinching. . . . 3rd. They come on after some physical or moral shock, and often present strange contrasts to the previous habits of thought, or have no relation thereto. . . . . 4th. In many cases they have relation to the patient alone, and are often of a kind which renders their nature apparent." He properly states that these characteristics are not constant. But we believe that it will be found of great service to bear in mind this excellent analysis of insane thought in investigating doubtful cases of insanity.

This is also the place to introduce Dr. Williams' views of what constitutes insanity. This author, perhaps warned by the Icarian fate of those whose too venturous wings had carried them into the deceitful sun of definition, gives us no precise exposition. Taking a less ambitious course, and keeping nearer to the every-day world of human thought, he seeks for no general expression that shall include all the insane and exclude all the sane. Looking at the question merely in a practical light, he adopts the invaluable test of Gooch, who, declining all generalities, makes the peculiarities of the individual the great object of investigation. "A particular act, or succession of acts, to acquire value as a symptom of insanity, must do so through the fact of its denoting a departure from the natural and healthy character, temper, or habits. It is not, therefore, sufficient that the medical man who would determine the question of soundness or unsoundness of mind be informed of special acts which he contrasts with what he may regard as an approved standard of natural health; but it is requisite that his standard be the admitted mental health of the individual, that the acts specified may have their value determined accordingly; since, to quote the words of Dr. Combe, 'it is the prolonged departure, without adequate external cause, from the state of feeling and modes of thinking usual to the individual in health, that is the true feature of disorder in mind.'" We have only to object to this that the words, or, more properly speaking, the opinion attributed to Dr. Combe, belong to Dr. Gooch, who had developed and enforced the method of testing the individuality by itself, with remarkable felicity, in his celebrated article on the case of Mr. Davies, in the *Quarterly Review*.

We have always thought the following anecdote of Charles V. to be an admirable illustration of the futility of general definitions of insanity, and of the necessity of judging of every individual by his own standard. In his retirement from the world this celebrated man was accustomed to employ his leisure in forming curious works of mechanism; and he was said to have been particularly curious with regard to the construction of clocks and watches. He found, after repeated trials, that he could not bring any two of them to go exactly alike, and hence was led to reflect, with a mixture of surprise and regret, on his own folly in having bestowed so much time and labour on the more vain attempt of bringing mankind to a precise conformity of sentiment concerning the intricate and mysterious doctrines of religion. "Tot hominum, tot sententiarum;" and "tot hominum, tot mentium."

The whole treatise of Dr. Williams is a most able, searching, and philosophical analysis of the question of what constitutes insanity. So able, indeed, is it, and so consecutive in its reasoning, that we find the utmost difficulty in making extracts in such a manner as to exhibit a fair and adequate idea of the character and scope of his argument. We have, however, stated in this place his leading principle as bearing upon the argument we have taken up, and hope to find other opportunities of developing his views as we proceed.

What we have to object to Dr. Mayo's test of insanity is, that it is not a medical test at all: it is metaphysical and legal. And, to describe it more closely still, it is a purely intellectual test. We do not call to mind any physician of repute in psychology, who maintains the validity of such a test in the absolute form in which Dr. Mayo states it. Perhaps the nearest approach to it is the definition of Dr. Conolly, who places the fundamental criterion of insanity in the "comparing faculty." That he has legal authority, we will not deny. He may cite, for example, the dictum of Baron Alderson, who, in the case of Robert Pate, said, "In the first place they must clearly understand that it was not because a man was insane that he was unpunishable; and he must say that, upon this point, there was a very grievous delusion in the minds of medical men. The only insanity which excused a man for his acts was that species of delusion which conduced to, and drove him to commit, the act alleged against him. They ought to have proof of a formed disease of the mind—a disease existing before the act was committed, and which made the accused incapable of knowing, at the time he did the act, that it was a wrong act for him to do." Here we have judicial authority for Dr. Mayo's favourite doctrine of insane responsibility, and for the sufficiency of the test of delusion. But we conceive, whilst expressing and entertaining a just and great reverence for the learning and integrity of the men who so worthily fill the seats of judgment in this country, that we are under no necessity, at this time, of refuting maxims and assumed rules of practice which the judges themselves had hardly uttered before they were found acting in irreconcilable contradiction with respect to them. We are amply justified in saying that the judges who gave their exposition of the law on this subject in a deliberate Report to the House of Lords, have themselves, in practice, abandoned their own exposition; and have, in many remarkable instances, been content to accept the skilled testimony of medical witnesses, in arriving at their decisions as to the essential conditions of insanity. We will not, therefore, stop to exhibit the impracticable fallacies of legal definitions. They have been often exposed in these pages, and by many medical writers; but by none better or more conclusively than by Drs. Bucknill and Williams in the works before us. We decline, therefore, to accept from Dr. Mayo the testimony of lawyers upon this matter. As a physician, we look to him for medical reasoning; unless, indeed, what seems not very remote from the drift of his argument, he is prepared to give up the insane altogether to the law.

It is scarcely necessary now, to prove that delusion is altogether untrustworthy as a test of insanity. In fact, it is as difficult to define insane delusion as it is to define insanity. Obscurum per obscuriùs. That admirable physician, Leuret, has well said he never met with an idea in a lunatic asylum, howsoever preposterous, extravagant, and unnatural, which he had not seen matched in the world. Insanity, then, cannot consist in the idea. To centre all insanity in the intellect, is to keep out of sight that inseparable part of the human mind, the moral element, which is so often the source, *quoàd* the mind, of intellectual disorder. Dr. Mayo first discards disease as a necessary condition in insanity; at another part of his work he even contends that the *mind may be diseased in the abstract.* But he goes further: not content with loosening the body from the mind, he next amputates, if we may use the expression, one part of the mind from the rest; he cuts off the moral element from the intellectual, and thus reduces man to a being without emotions, without passions, or at least, without any right to have them. Such a man has clearly no business to become insane; and, certainly, since he cannot attribute his intellectual aberration to disease, nor to disorder of the moral element, he has no right to plead insanity in bar of punishment for any act that he may commit. It is unfortunate, however, that the human intellect is cemented with earthly dross; and that the faculty of rendering the mind independent of the actions of the body, is one after which the philosopher may sigh, but which the frailty of man cannot reach.

Dr. Bucknill has dwelt ten years in an asylum. He has conducted, with more than common skill and minuteness, necroscopic researches upon the bodies of the insane. He has brought an acute and philosophical mind to work upon his observations of the wanderings of the insane mind, and of the abnormalities of the physical structures. It would be strange if he were to believe in the independency of the mind upon the body. He speaks from an authoritative experience, when he declares his conviction that insanity invariably implies disease of the brain. And we avail ourselves of this opportunity of recording our opinion that he has rendered an eminent service to general pathology and to psychology, by his admirable investigations of cerebral disease, by observing the variations of the specific gravity of the brain in the insane. His results confirm in a conclusive manner the observations of Ferrus and Guislain, that atrophy of the brain is a frequent condition in many forms of insanity.

But, although Dr. Bucknill avoids the flagrant heterodoxy of Dr. Mayo, we are unable to accept even his definition without reservation or comment. "A false action of conception or judgment (intellect), a defective power of the will, or an uncontrollable violence of the emotions and instincts (moral), have *separately or conjointly* been produced by disease." He thus admits disease, and does not exclude any one of the integral parts of the mind. But, if it be fair to use an author's own arguments against himself, we will point to Dr. Bucknill's theory of the emotional origin of insanity (p. 83),—a theory forcibly expressed by Guislain—and ask how this theory can be reconciled with the above definition, and with the explanation of it, in which he says that pure and unmixed cases of Intellectual, Emotional, or Volitional insanity may be found? Is not this to say, insanity may be not only emotional, but intellectual or volitional in its origin? But, in another place (p. 85), he says, "The will is a faculty so simple and undecomposable that *it may well be doubted if it can ever lapse into a diseased condition.*" Shall we, then, exclude volitional cases of insanity? If we do so, Dr. Bucknill's definition will be condensed to intellectual or emotional impairment produced by disease. Now, is it true in nature, that *pure* cases of either intellectual or emotional disease exist? Is it true that the intellect and the emotions can be isolated from each other in disease any more than in health? Is it not, rather, true that there are cases of insanity in which the prominent or most obtrusive character is aberration of the intellect, or delirium, and others in which the most obtrusive character is abnormality of the moral part? But does it follow that, because one of the integral parts constituting the human mind is strikingly deranged, the other is altogether sound? We, for our own part, demur to this conclusion; and beg to refer to a passage in which we have on a former occasion embodied our ideas upon this subject:* "that as the mind can be only occupied with one idea at a time, it is, as a *whole*, affected when under the influence of

* *Psychological Journal,* vol. v., p. 466.

any specific lesion." We may say, further, without losing sight of the fact that the mind, although so constituted as to present different phases, which appear to be distinct parts, that it is in reality one and indivisible. We are disposed to regard the intellect as the highest mode of mind, rather than as a distinct component. Lower modes of mind are evinced in the active and moral powers, including the appetites, desires, affections, emotions, and the moral faculty. It is out of these last that spring all the great motions of thought and action. The intellect takes its form and direction from these. In the *mens sana in corpore sano*, a strong intellect may control every inordinate impulse, although it cannot extinguish them. In the *mens insana in corpore insano*, the primum mobile, that part from which actions take their spring and the intellect its bent, dominates over the intellectual faculties, which often struggle long but ineffectually against the morbid suggestion. It is possible, then, to imagine a diseased mind—using the term as meaning a mind alienated through disease of the physical structure—in which the intellectual aberration shall be with difficulty or not at all recognised, in which there shall be *no* delirium, but which shall, nevertheless, be under the dominion of abnormal moral powers. That such is indeed the case, the records of science and the experience of psychopathists abundantly prove. And, since the abnormality or disease of the moral powers, in many cases, only finds its exponent symptoms in the phases of the intellect, we can understand how difficult it often is to detect some forms of moral insanity. In the most perplexing cases of all, the expert has little open to him beyond the ordeal of interrogation. But, every articulate speech implies exercise of the intellectual faculties. These, aroused by the necessity of replying to questions, often come to the aid of the monomaniac, who, driven to use them under the controlling influence of observation, keeps his moral part in subjection for a time, and makes no sign that reveals his disorder. Again, as in many bodily diseases, as in ague and epilepsy, for example, although we cannot doubt in these a persistent morbid action, the frame is not at all times fevered or convulsed, so in mental diseases, the persistent moral lesion may not always be in the ascendant to such an extent as to overpower the intellect and lay itself bare to the world.

But these difficulties are *difficulties of diagnosis;* and ought not in reason to be interpreted as proof against the essential *existence* of disease.

We recognise, then, a form of monomania, not in the pure etymological signification of the word, but in that practical sense which is understood to represent what we actually find.

The moral part diseased, and the intellectual part sound and intact, is a condition that the human mind perhaps never presents. In such a sense we should deny that moral insanity, as a disease, exists. Show us a man whose moral you say is diseased, but whose intellect is always clear and uninfluenced by his moral abnormity: and we will answer that since it is the function of our intellect to weigh what is wrong against society, and to direct aright the actions prompted by the moral, that man is responsible for his conduct to his fellow men; but we deny that such a man is insane; we deny that he is diseased.

We have thus largely expressed our ideas upon this question of the nature of insanity, because it is applicable in order to show upon what our objections to the definitions of Dr. Mayo and Dr. Bucknill rest. It is, at the same time, anticipative of what we might otherwise have to say upon monomania and moral insanity, in commenting upon the views of our authors upon these subjects. When we arrive at this point of our task, we shall be able to refer our readers back for some of the reasons we have to urge against the views of Dr. Mayo and Dr. Bucknill, to what we have just written.

The views of Dr. Mayo and Dr. Bucknill on the much-disputed subjects of monomania and moral insanity, may be, to some extent, inferred from their general expositions of insanity. The first conclusion which Dr. Mayo refers to in his preface as one of his "*adventurous* speculations,"—an epithet the correctness of which it is not for us to dispute —"concerns the ambiguous, and, as I think, mischievous nature of some doctrines, suggested by the term 'moral insanity,' or certain synonymous expressions." He who contends that delirium, the evidence of disordered intellect, is the essential test of insanity, necessarily, as we have seen, denies the existence of moral insanity. In the absolute sense, we have ourselves already said that moral insanity does not exist; but Dr. Mayo's commentary—in some places not without force —upon the cases and arguments of Pinel and Pritchard, goes much farther than this.

"I have," says Dr. Mayo (p. 53), "now to consider a peculiar state, under which the leading and important subject for judicial consideration is an orgasm, or an intense and sometimes sudden desire, which leads the sufferer to perform some criminal act; this orgasm not always susceptible of being construed into delirium, as not being obviously attended either by a morbid delusion or by a state of inconsecutive thought. In regard to this condition of the case a question instantly arises, whether in the absence of direct, it may admit of constructive proof of delirium,—that is, of a morbid state of intellect; or must be removed into the category of vice." He admits that there are some "cases of the above orgasm in which it seems to suggest to its victim an objective false perception, leading him to some criminal act to which all his feelings and moral sentiments are opposed." Thus, when delirium is made out, Dr. Mayo would not deny a form of insanity which is usually designated as moral. But this evidence he must have, or he will allow no insanity. We must place an illustration before our readers (p. 59): "With respect to the misapplication of the plea of insanity to hysteria, we have a case of a nursery-maid, placed in Bethlem Hospital in 1846. A trifling disappointment relative to an article of dress had produced in her a wayward state of mind. She laboured at the time under diminished catamenia. An object to which she was generally much attached came in her way,—namely, the infant whom she nursed; and she destroyed it, as a fanciful child breaks, in its moodiness, a favourite doll. No fact more nearly approaching to delirium than the above was stated in exculpation or excuse at the trial. But Dr. Prichard's work was published in 1842, and by 1846, juries had learned to convert the uncontrolled influences of temper into what he terms instinctive insanity."

In reference to such a case as this we will remark that those who, like ourselves, have studied with care the influences which ovarian and uterine disorders exert upon the nervous system will easily call to mind cases without number in which such disorders, more especially those in which hysterical convulsions recur at the menstrual epochs, clearly affect the mind, both in its moral and intellectual phases, in a degree that borders upon, and not seldom amounts to, temporary insanity. We wish particularly to observe, that the mental alienation in these cases is, like the bodily disorder, of a paroxysmal and periodical character. In the intervals between the accessions an ordinary observer, or even the physician, would not detect in the conduct or speech of such a patient any aberration. He would scout the idea of insanity. Very often, also, the true and perilous condition of the patient will not be revealed by word or deed, even at the acme of the physical and mental disorder. Conscionsness of her danger, and of her want of control at these seasons, will sometimes warn her to take certain precautions, and place a guard over her utterance and actions, when the period of danger is imminent. But, nevertheless, the danger is really and terribly present. From what we have observed in some patients of this kind, who, on common occasions and under the controlling influence of the observation of strangers, would pass for persons endowed with the healthiest mental organization, we should be surprised at no act which they might commit under the circumstances described. But these circumstances of abnormal ovarian and uterine function or their bearing upon her mental health, might be unknown to, and withheld from, the physician who may be consulted as to the nature of an apparently criminal act she may have committed. When brought under his observation, the insane fit, the "orgasm," may have passed over: the ovarian excitement has subsided; the nervous commotion is subdued; no delirium remains. Judged by her actual condition she will be pronounced of sound mind; but a fatal error may have been committed, through ignorance. Taken at this time, and upon the evidence alone which this supplies, she would, if Dr. Mayo were consulted, be condemned to expiate a deed committed when insane by undergoing the full rigour of the law. But Dr. Mayo asks, if there may not be "constructive" evidence of delirium at the time of the act. We will, therefore, go back to the period of the fit. How shall we arrive at constructive evidence? Will Dr. Mayo accept the constructive evidence derived from the presumed criminal act, or from other acts that took place at about the same time? We fear not; and yet there may be no other evidence. We think we do not impute to him what is not the natural conclusion from his reasoning, when we assume that he would repudiate all construction of this kind derived from the alleged criminal act itself. And if he reject this—presumably, at least, the most irrational, the culminating act—he must needs reject those lesser acts that were committed about the same time. Besides, we do not know that Dr. Mayo would admit that action alone may indicate delirium. And yet it is impossible to forget that action may be as much an expression of the human intelligence as speech. The deaf and dumb may be insane, nay, brute animals may be insane, and not unfrequently are so. The probability of such cases Dr. Mayo would probably not deny, but we do not see in what manner, following his principles, he would obtain evidence of delirium. We will, then, seek for this evidence in speech. The typical patient we have supposed may, although possessed of the faculty of speech, have been placed in the position of the dumb madman. During the transitory paroxysm, no one may have been nigh to hear her, or the alienation itself may have been of a nature to explode in action and not in words. There may then have been no evidence, direct or constructive, of delirium such as, if we may judge by the case we have quoted from him, would satisfy Dr. Mayo. But this unhappy girl may, nevertheless, as we have shown, have been insane; there were persons of skill and judgment who believed that she was. Can Dr. Mayo, who feels so much repugnance to admit that she was insane, *prove that she was not?* What is the alternative in such a case? If we adopt the opinion of those who affirm insanity, detention in an asylum is the result; if we adopt Dr. Mayo's denial of insanity—the actual insanity, we have a right to assume, remaining the same—the gallows would cut short the dispute. According to the first position, society would, in the event of error, receive no detriment; according to the second, humanity would be outraged. We acknowledge the difficulty—the sometimes insuperable difficulty—of arriving at conclusive evidence in cases of this nature. But difficulty of diagnosis, we say again, is no argument against the possibility of the existence of a disease. It is enough for us that such disease as we have described may and does exist. This admitted, in cases of extreme doubt, not humanity only, but science and justice, demand that our decision turn to the side of mercy.

And if we pursue the argument of Dr. Mayo, we shall find that even he supplies the materials for his own refutation. At p. 63 he quotes the case of Mendic from Georget:—" Hypolite Mendic, a non-commissioned officer in the French service, had gradually become morose, capricious, and brutal in his conduct, so as to excite the disgust of all his companions. This ends in disobedience of orders, and such violence towards his commanding officer as to render him liable on trial to the sentence of death. The trial proceeds *with the customary anxiety of the medical witnesses to make out a plea of insanity*, but in the course of the trial one weighty fact was made out,—namely, that before his outbreaks he was subject to an epileptiform seizure, out of which he emerged into the wayward state above noticed. This might fairly justify an hypothesis of delirium, as present at those paroxysms."

Now, we submit that if the hypothesis of delirium—if nothing less than that will satisfy Dr. Mayo—or of absolute insanity be fairly justified on proof of epileptiform seizures, then by parity of reasoning a similar hypothesis is justified in the case of the girl who was affected with hysteria and disordered menstruation.

On this subject we are happy to find both Dr. Bucknill and Dr. Williams agreeing with us on the leading points. Dr. Bucknill says with much truth:—" The doctrine (p. 82) has, to some extent, suffered both from bad terminology and from bad logic on the part of its advocates, and especially from its having been considered separately from the necessary and essential requisite of irresponsibility, *a state of disease*." We are very much inclined to recognise the justness of Dr. Bucknill's objections to the terms "uncontrollable" and "impulse," reserving always our opinion that the insane condition usually understood when we use those terms has a real existence. "The term impulse," he says (p. 84), " conveys the idea of force communicated instantaneously, a rapid motion; whereas the morbid desires under consideration are not of instantaneous production or of rapid growth. They arise from a chronic disease, and are resisted up to a certain point."—"The adjective (p. 85) *uncontrollable* is also liable to serious objection."—" The real question is not whether the emotions occasioning the overt act are beyond the power of the individual to control, but whether they are the result of disease." We have only to add to this the qualification that the form of insanity commented upon is not always of slow growth, or dependent upon chronic disease; or if it be, that the duration of the antecedent disease is in some cases to all evidence short; a reservation must also be made for those cases depending upon morbid ovarian irritation to which we have alluded.

Dr. Bucknill suggests the following modification of Esquirol and Marc's classification of homicides by the insane:—"1st. Those wherein the crime has been occasioned by delusion, and no reasonable person can doubt or object to the irresponsibility of the offender. 2nd. Wherein the offender, through suffering from mental disease, has committed the crime under the influence of some motive not of a delusive character. Such are the cases of Lawrence, Touchet, Hadfield, Greensmith, Staniought, Burton, and others. In these cases, the responsibility may be diminished or modified, but the most extended sympathy for the insane could scarcely claim for them that it should be altogether abrogated. 3rd. Where with general symptoms of cerebro-mental disease, neither delusion nor motive for the crime are discoverable. These latter are the cases which with a most unlucky phraseology have been attributed to *moral insanity*, insane impulse, uncontrollable impulse, homicidal impulse, &c."

Dr. Bucknill does not then appear to deny the actuality of those forms of mental disease commonly, although perhaps erroneously, called moral insanity; but he doubts whether, whilst admitting them to arise from disease, they ought to confer complete irresponsibility. Indeed, looking back to his definition of insanity, it is clear he is precluded from denying the existence of moral insanity. Upon the question of gradations of responsibility we shall presently have to offer some remarks.

The great question of moral insanity is investigated by Dr. Williams in the most masterly manner. A tone of thoughtful and unprejudiced reasoning pervades his elaborate and logical analysis. We again express our regret that we cannot, even by means of long quotations, convey to our readers such a digest as would not break the chain and mar the force of his argument. It is our duty, however, to place his leading conclusions upon this subject side by side with those of Dr. Mayo and Dr. Bucknill. He thus clearly lays down the problem to be solved, (p. 80., *Dublin Quarterly Journal*, No. XXXVII.):—"If then, in the commission of crime, neither the moral nor intellectual principle appears to act independent of the other, let us inquire how far they are identified, and to what extent their unity is involved, when resulting in the exercise of acts open to the charge of criminality." He thus contends against *pure* moral insanity:—"If we go to the full extent of some writers, and allow the moral intelligence to be, *per se*, diseased, or '*Manie instinctive sans délire*' to be present, while the reasoning powers are *wholly* unaffected, what else can we suppose but that every case of confirmed viciousness is an example of such a form of disease?" At p. 279, No. XXXVI, he describes or defines monomania "as a disease in which the mind appears to suffer from a paralysis of its powers of conception, and is inadequate to appreciate the general or partial relations which the subject-matter of the monomaniacal conceptions involves." This view coincides closely with that of Guislain and Renaudin. He says:—"All examples of moral insanity which the records of criminal jurisprudence supply, may be ranged under one of the following heads: I. Cases in which the development of the moral feelings or affections appears as originally deficient. II. Cases in which the perversion of the moral feelings or affective faculties appears to occur incidentally. III. Cases in which the moral feelings appear to become universally disordered. IV. Cases in which the moral feelings appear as partially diseased." Dr. Williams thus sums up his conclusions:—"I. By the term moral insanity, we express mental unsoundness, chiefly evidenced through the moral or affective faculty. II. Though the moral and intellectual perceptions are capable of independent exercises (?), yet in their effective operations they mutually blend together and co-operate. III. Analogous differences are observable in the development of the morals of the intellectual faculties. IV. Those differences are such as seem to impart a special character or disposition to each. V. The intelligence is, in the healthy, properly regulated mind, capable of controlling and directing the moral exercises. VI. The moral or affective faculty being closely associated with the sensational, is, therefore, in nearer relation to the personality. VII. Disease, affecting the personality, may occasion morbid changes in the moral disposition without immediately involving the intelligence. VIII. From the intimate blending, intricate co-operation, and mutual dependency, of the separate mental faculties, causes producing abnormal action in the one, unusually, (usually ?) eventuate in causing derangement of the other. IX. Though in derangements of the mind the moral faculty appears primarily and solely involved in many instances, the non-development of intellectual unsoundness, through other manifestations, cannot be received as proof of its non-existence. X. The sense of moral perception is found to vary according to the nature and extent of the guidance it may have received. XI. The moral faculty, although incapable of determining positive duties, is adequate to oppose intellectual suggestions in such exercises as now immediately involve the moral perceptions. XII. A want of accordance between the moral and intellectual faculties, may proceed from either undue excitement of the moral or inefficient exercise of the mental powers. XIII. Those causes adequate to affect either faculty must be carefully sought for, previous to offering an opinion. XIV. That as those causes involve questions of a physiological, pathological, and strictly medical nature, irrespective of their ethical, logical, or legal relations, their proper estimation requires such a combination of knowledge as none other than a psychological physician could be reasonably expected to possess." We commend these propositions, which embrace the elementary principles whence the existence and nature of moral insanity may be deduced, to the careful consideration of our readers.

We pass on to the weighty practical question before us, the conditions of responsibility, as applying to insanity in general, including the forms distinguished as moral insanity. And, first, let us state how our authors would respectively define those conditions. Dr. Mayo says: "No abnormal state of mind confers irresponsibility (an attribute which the inventors of the term 'moral insanity' conceive it to possess), unless such abnormal state of mind involve intellectual as well as moral perversion." We have already seen that, to constitute intellectual perversion, Dr. Mayo requires proof of inconsecutiveness or of delirium. From the headings of his third lecture we gather the following:—"That idiocy (!) and unsoundness of mind (a condition Dr. Mayo endeavours to discriminate from insanity) each involves some responsibility in reference to crime." He does not state or give any rules for estimating the degrees of responsibility these states involve; his definition of insanity even, being made to turn entirely on delirium, utterly failing in enabling us to determine who is sane and who is not; even if we could determine, by his test, who were insane, we should still have to seek for new evidence to determine the degree of responsibility (for the insane are not irresponsible), and yet, leaving the question thus vague, we are told by Dr. Mayo (p. 87), "Meanwhile we cannot urge that a scale of secondary punishments is involved in any insurmountable difficulties of application"!

Are Dr. Bucknill's statements more precise and satisfactory? He thus lays down his limitation (p. 15): "Insanity being a condition of partial change, it is difficult for the psychopathic physician to deduce from it the result of total irresponsibility. Logically, the loss of responsibility must be held to be co-extensive with the amount of disease." But how is this amount to be ascertained? At p. 59 he says: "*Responsibility depends upon power not upon knowledge, still less upon feeling.*" We find nothing much more precise than this. It is often difficult, as we too well know, to prove actual insanity; but what is this difficulty compared with that proposed to us of constructing a graduated scale of disease and responsibility, which, to be just or applicable, ought to be as nicely divided as the scale of a thermometer! Until we are supplied with the necessary psycho-pathometer we fear we must be content to adhere to the old-fashioned and intelligible rule, that insanity confers irresponsibility.

We gather from Dr. Williams' treatise that he also would consider a modified responsibility of the insane proper, but we are unable to extract any clear or precise expression of his views upon this question.

We have, probably, already said enough to show the fundamental error and impracticability of this doctrine of the responsibility of the insane, but, before proceeding to another branch of our critique, we think it desirable to add a few more considerations. We cannot forbear quoting the just and humane reflections of Dr. Pritchard: "In the instance of instinctive insanity, or insane impulse to commit acts of violence and atrocity, to play the incendiary, or to violate the good order and decency of social life, it is obvious that the only thing requiring much consideration is the real existence of disease, and its distinction from ordinary and real criminality. . . . Whether he ought (disease being proved), in any case, to undergo other punishment (than seclusion) is a question which I do not feel disposed to discuss. As we have seen that a struggle has often taken place between the desire to commit any violent act and the conscientious feelings of the unfortunate person who is thus tempted, it is probable that some have yielded to the temptation, though convinced that they ought to have resisted it. Such persons must be admitted to be morally guilty, and to deserve to suffer. But the calamity with which we know them to be afflicted is already so great, that humanity forbids our entertaining the thought of adding to it. Perhaps all that we ought to aim at in such a case is, to secure the community against the evils to which it may be exposed."

Dr. Bucknill argues that the degrees of responsibility and of insanity stand in an inverse ratio to each other. What is this but to say—since no man is wholly insane or demented—that *no* madman is wholly irresponsible? To carry this proposition out to its logical conclusion, as it ought to be if the *principle* has a foundation in truth, would, therefore, be to bring every insane person under the penalties of the law. The plea of insanity would no longer be admitted, as it is now admitted, to exempt a person from trial. According to Judge Hale all criminals were insane: Dr. Mayo and Dr. Bucknill reverse the proposition, and say, all insane are criminal. According to them insanity ought not to be pleaded as a ground of exemption from trial. They would have the jury first determine whether the crime imputed has been committed, when the rational idea of crime is an absurdity, a self-evident contradiction, and then to investigate the question of insanity; and then—is not that enough? No! the jury must then make out the *degree* of insanity! They must apply the yet-to-be-discovered psycho-pathometer to determine the exact measure of responsibility, and then — climax of contradictions, the judge must adjust his scale of secondary punishments, admitting the insanity as an "extenuating circumstance!"

Is it necessary to observe that this doctrine involves an entire subversion of the relations and duties of medical and legal practitioners in cases of insanity? Prisons must be substituted, to a great extent at least, for hospitals; or psychopathic physicians must become gaolers. Dr. Bucknill and Dr. Mayo would consign one of their "responsible" lunatics, if there is any meaning in this argument of secondary punishment—we presume they would not hang the very worst of them—to incarceration, as a *punishment*. Who, we ask them, is to be his custodian? Is he to be sent to an ordinary gaol? But they admit he is diseased; and who shall heal the sick but the physician? Dr. Mayo, who holds the eccentric idea that the "*mind may be diseased in the abstract*,"—a notion we shall presently discuss —would probably send him to *gaol* to be lectured to by the chaplain, and disciplined by the turnkey! But Dr. Bucknill would, as we interpret his views, send him to a special asylum for "criminal lunatics." He would, therefore, place them under the care of a physician; but the profession and duties of a physician forbid him to be an officer for executing the final sentences of the law. Dr. Bucknill is a physician, and an able one, not a gaoler: his "responsible lunatic" would, therefore, come under his care as a *patient*, not as a *prisoner;* as one requiring *medical* not *penal* treatment. And if this be so, is not the whole case conceded, namely, that no insane person who is a fit subject —as we presume all insane persons are—for *medical treatment* can, with propriety, be made the *subject of criminal punishment?*

Shall we point to yet another subversion of all intelligible principles of sanity and insanity necessarily involved in this doctrine? Shall we ask what it is that constitutes insanity; is it the disease, the antecedent morbid lesion of the body entailing disorders of the mind, or is it the overt act of violence or wrong that makes the madman? Dr. Bucknill expressly contends that, even in the most difficult cases, namely, those of moral insanity, the terms "uncontrollable impulse" are improper, because the act was owing to "chronic disease." What, then, is the overt act, but the accidental culminating point of disease, of insanity? Now, being diseased, the patient ought to be, and would have been, had his malady been detected in time, placed under medical care before the perpetration of the overt act. Is society to take advantage of its own *laches* and *punish* a sick man for that act which emanated from his sickness, and which ought to have been anticipated? Who can estimate the number of deeds of violence which are annually prevented through the medical treatment of insane patients? Are they insane whilst under control and protected from committing deeds of violence; and do they only become criminal and responsible when left to themselves, with free scope for their disease to declare itself by deeds of violence?

But we have surely said enough upon this subject to leave it with confidence to the decision of every man who is willing to reason from facts, the constitution of the human mind, the first principles of justice, and to every physician who has not a theory to maintain, and who is not given to indulge in "adventurous speculations."

But we have now to exhibit another doctrine of Dr. Mayo's, which, as it is not classed with his three "adventurous speculations," we presume, so commends itself to his judgment as not to require any particular apology in introducing it to his readers. At p. 24, we find the following passage:—"That there should be a *disease of the mind in the abstract*, that such disease should work changes in us, viewed in this light, analogous to the physical changes of our bodily organs, is neither unnatural nor inconceivable (?) A parasitical growth—if for want of a proper term I may borrow this epithet from physical speculation—may take place under such disease, itself possessing vital functions and energies (!) but having no other relation to matter than the obvious one on which the tenure of our present life is based,—namely, that we have an immaterial and a material being indissolubly bound together for the duration of that life, while, for anything we know, the immaterial element may be just as subject to its proper affections as the material one is. . . . . The above remarks may at least have a wholesome tendency to keep before us in our speculations *the immense fund of mental disease that may exist*, inappreciable through any knowledge that we at present possess of phenomena so little capable of being made the subject of experiment, or even observation, as those which I am SUPPOSING." (!)

And this is *not* an "adventurous speculation!" It is "neither unnatural nor inconceivable that there should be disease of the mind in the abstract!" Nay, more; it is "neither unnatural nor inconceivable" that a new immaterial entity, "a parasitical growth, itself possessing vital functions and energies," may take place under this abstract disease! He borrows this image, this "parasitical growth," from "physical speculation," and it does not check his "adventurous speculations" to find himself unable even to conceive of mind at all without having recourse to physical ideas! But having thus set up this ideal "parasitical growth," Dr. Mayo straightway applies his discovery, as if it were an established fact in psychology, to the practice of medicine, the administration of the law, and the relations of society! It was said by Locke, and its truth is generally recognised, that we arrive at our knowledge of the human mind in general by reflecting upon the operations of our own. But it is clear from this mental experience of Dr. Mayo's that we must receive the speculations even of the greatest *abstract* philosophers with considerable caution. Dr. Mayo, we may presume, discovered this "parasitical growth" in his own mind; at least there is room for reasonable doubt that it exists anywhere else; and we should be slow to admit this speculation, although not "adventurous," nor "unnatural," nor "inconceivable," so long as it remains the undivided and characteristic property of Dr. Mayo, as a general fact in the constitution of the human mind.

But we should think this speculative abstract insanity unworthy of a moment's serious attention, but for the light it throws upon the author's conclusions about insane responsibility and the alteration of our mode of criminal procedure. We must therefore examine it a little more closely, and we take leave to ask Dr. Mayo how it was that, taking no account of the "parasitical growth," he became possessed of evidence that the mind can be "diseased in the abstract"? Has he enjoyed the singular privilege of observing the mind in the abstract in any condition, healthy or diseased? Many superstitious persons are still found who believe that disembodied ghosts exhibit themselves upon earth, — and some are said to play strange freaks, — but has any one yet seen or described *an insane ghost?* Certainly no one, unless it be Dr. Mayo. But there is another condition of mingled superstition and imposture under which it has been held that the mind can be separated from the body. Is Dr. Mayo a mesmeric philosopher? Does he possess the faculty of "projecting his mind" from his body, and so of studying it in the abstract? If it be in this manner that he has arrived at his conclusion, we must imagine that he was operated upon by a neophyte in the art, and that under unskilful "passes" poor Dr. Mayo's forsaken body did not succeed in getting its mind back again entire after its temporary divorce. If this speculation be correct—and it appears to account for the looseness of Dr. Mayo's theory—we should urge him to place himself under the hands of a more expert performer, to "project his mind" again in search of the part that was lost, and to take the opportunity of dropping that "parasitical growth" which it was so unfortunate as to pick up in its first expedition.

We are really anxious to know how Dr. Mayo has penetrated beyond the boundary of the material world, and so we will ask him another question. Is he a secret disciple or adept in the doctrine of Veda, who, although not like Berkeley, denying the existence of matter, yet claimed for his followers the power of excluding all ideas derived from the external world, and the capacity of arriving at a purely spiritual existence? If Dr. Mayo believes in ghosts, mesmerism, or the doctrines of Veda, we trust he will exhibit the courage of his opinions, and declare himself; or if he believes in none of these, we submit that he is bound to inform the scientific world by what other means he has acquired the conviction that mind may be diseased in the abstract. Were the questions we are now considering such as interested physicians only, it would be impertinent on our part to point out by a single serious argument the necessary dependence of mind upon body. But they have an immediate bearing upon legislation, and are therefore discussed with eagerness by lawyers and all persons of education. We think it, therefore, useful to state that it is a matter of chemical and medical observation and inference that no exercise of the mind can be carried on but at the direct expense of physical matter. Let a man endeavour as he will to lose sight of his body, and to indulge in the purest spiritual reveries that he can, the result of his spiritual abstractions will be felt in the waste of corporeal tissues; brain-matter has been used, and will have to be repaired. So impossible is it even to conceive at all without the participation of the body, so idle is it to talk of insanity in the abstract!

But, however arrived at, Dr. Mayo enunciates this hypothesis. Having adopted it, some of his medico-legal conclusions will appear the less extraordinary. For example, he who argues that the mind exists as a separate entity from the body, or that it can be investigated in any other way than through physical manifestations, and that it is liable to become diseased *per se*, without respect to the body, is of course at liberty to contend that an insane person is responsible to the law, although *we* find it difficult, even from this extravagant premise, to deduce this equally extravagant conclusion. For whether the mind originate disease in itself, or acquire it from the body, is there not still disease that destroys power and responsibility? But Dr. Mayo, with stoic sternness, ever eager to bring the lunatic within the gripe of the law, advocates another doctrine which also hangs upon the theory of abstract mind-disease. He contends, of course, for responsibility under so-called "lucid intervals." We are told (pp. 49, 50) by Dr. Mayo, in combating the able argument of Ray—who with unaccountable illiberality is not permitted to retain his name as it came to him from his father, and is given on his title-page, but who is always styled by Dr. Mayo, Dr. *Rae*—"Esquirol found that out of 2814 recoveries of the insane, 292 have recurrences of the disease. These persons, then, had obtained temporary cures, and I know not how Dr. *Rae* could refuse to any criminal outbreak of which they may hereafter be guilty, the same immunities from punishment, and on the same grounds, which he confers upon persons presumed to labour under the temporary recovery afforded by a lucid interval."

From this passage it is clear that Dr. Mayo does not know the difference between a "lucid interval," the very expression implying a continuance of disease, and a recovery, otherwise he could not apply the same reasoning to both conditions. Things opposite in their nature are not the subjects of analogical reasoning. It is necessary for us then to point out that all the most distinguished pathologists of the present day maintain that a "lucid interval" in the sense of absolute temporary recovery, the sense in which lawyers and Dr. Mayo understand it, does not exist. The supposed "lucid intervals" can only be regarded as *remissions* of the disease, the disease, like ague or epilepsy, subsisting nevertheless. Is a man free from ague, or healthy, during the intervals between the fit? Is a man liable to periodic attacks of mania, separated by intervals of comparative quiet, a healthy man? Such a notion can only be maintained by metaphysical speculatists, who dream of mental disease in the abstract. It is contrary to all sound pathology, and no less subversive of justice and humanity.

It is beyond our text on this occasion to discuss the treatment of insanity, but we cannot help asking, what hope of successful treatment is held out if we adopt Dr. Mayo's abstract speculations? Few will share his expectation that the dread of the rigour of the law, or the exhortations of ministers of religion, would exert either a curative or a preventive agency, and still fewer will look with favour upon a theory which, carried to its natural conclusion, would discredit altogether the intervention of the physician in the cure of the insane, lead to the demolition of the county lunatic asylums, and the substitution of the turnkey, the gaol, and the hulks, and even the scaffold.

Is it necessary to pursue absurdity further? Shall we ask Dr. Mayo how he reconciles it with even his sense of justice, that the body which in a case of abstract mind-disease with "parasitical growth," must be presumed to be innocent of crime, should be condemned to suffer the penalties of the law for offences for which it could no more be answerable than the inanimate weapon which the maniac or the criminal employs? It is possible that the feeling and the judgment of the age are both erroneous; it is possible that Dr. Mayo, who we are to believe has seen much more than is dreamed of in our philosophy, is right. But we cannot regret that a decision based alike upon sound principles of philanthropy and sound observation of the phenomena of the human mind, is not likely to be overturned or disturbed by the baseless conjectures of men who substitute visionary and "adventurous speculations" for philosophical induction.

We pass on to the last question raised in the works before us. After divorcing mind from body during earthly life, Dr. Mayo proceeds to what, according to his own expression, is a *more* "adventurous speculation." His last step is to overturn the principles upon which the administration of justice is conducted in this country. Dr. Bucknill does not advocate the same measures as Dr. Mayo, but as we shall presently see, his propositions are scarcely more tenable or less dangerous. Dr. Mayo proposes to extend "to criminal cases that practice which actually prevails in analogous civil cases, as in commissions *De Lunatico Inquirendo*, of the examination of the party whose mental state is in question, in presence of the jury and the Court."

We scarcely think it necessary to dwell at any length upon the obvious objections to this proposal. What we have already said on the subject of secondary punishments for the insane, applies in great measure to this proposal to examine the persons pleading insanity before the court and jury. We will not stop to point out what must be obvious to every physician skilled in the observation of the insane, that such a plan is the very worst that could possibly be devised for the purpose of arriving at a just conclusion as to the sanity or insanity of a prisoner. We shudder at the thought of the horrors that might result from such a course. It often requires long observation under a variety of circumstances, of which privacy is the most essential, in order to form a satisfactory opinion as to the mental state of a person alleged to be insane. The force of the analogy from the practice in commissions *de lunatico* is more apparent than real. In many of these cases, the alleged lunatic is only present before the jury by a fiction; the inquiry in essential points does not differ from that in criminal courts; in both cases the jury *see* the alleged lunatic; they may hear him answer a few questions; but their decision turns upon the *evidence* of the medical witnesses who have seen and examined the person under proper conditions for forming their opinion. To examine in open court an alleged lunatic, who is really of sound mind, and who is accused of a criminal offence, would be in some cases to run imminent danger of discovering insanity where none existed; to examine in the same way a person really insane, would be a cruel and revolting proceeding.

Dr. Bucknill says (p. 120), "Of not less importance than some modification of the inflexibility of English law in relation to entire responsibility or irresponsibility, is the necessity of discovering some more fitting tribunal to decide upon the delicate question of insanity, than that rough instrument of justice, a common jury." He proposes "experts," who he says, quoting from Ray, "are persons appointed in the course of judicial proceedings, either by the court or by the agreement of the parties, to make inquiry under oath in reference to certain facts, and to report thereon to the court. They are not examined as witnesses, nor have they any power of deciding the cause like arbitrators; their functions are more analogous to those of a Master in Chancery. In reference to doubtful cases of insanity, their duties would perhaps approximate more closely to those fulfilled in our own Admiralty Courts by the masters of the Trinity Company. In intricate questions of collision, salvage, and cases of that nature, these experienced mariners are summoned to aid the court, as *amici curiæ.*" Dr. Bucknill fortifies his opinion by citing the following passage from the last Report of the Commissioners in Lunacy:—"If, upon the occasion of the trial of an indictment, the plea of insanity be set up, we are disposed to think that the question should be tried and determined by the court after taking medical and other evidence, and not by the common jury empanelled to try the facts."

In opposition to these suggestions, we might be content with stating the clear, common-sense opinions of Dr. Williams:—"Although we believe it to be essential that the physician have his mind thoroughly impressed with the true association between ethics and law, in order that he be the better enabled to estimate the question of mental soundness in its relation to crime; it is above all things important that in his professional opinions *he abstain from outstepping the bounds of medicine*, which freely consigns to juries the appreciation of the first, while equally denying their capability of adjudicating on the second."

We have italicised a sentiment in which we entirely concur, being convinced that nothing can tend more to lower the influence of medicine in the presence of the law than the practice advocated by Dr. Mayo and Dr. Bucknill, of trenching on the domain of either judge or jury.

"The ethico-legal considerations belong to the jury; the psycho-ethical to the physician."

"Society is fully warranted in being jealous of her rights, and equally justified in seeking to prevent any body of professional men from assuming an authority in reference to matters affecting her interests, those matters being within her own control. Both the bar and the public are, however, deceived when they presume the general not the particular application of medical opinions. The question to be determined in psychological investigations is not whether certain phenomena indicate the soundness of the mind or morals of all men, but how far they may enable us to estimate their relative condition in a particular individual."

Dr. Williams grapples directly with the question of medical juries: "If it be contended that medical men are so pre-eminently adapted for such intricate investigations, and it be conceded that cases may arise in which the physical estimate of crime involves many abstruse and difficult considerations, it may be asked, 'Why are other than medical juries empanelled to adjudicate on such matters?' To this we reply: There are many grave and fitting reasons that the existing state of the law should be maintained. Were medical men required to primarily decide on the soundness or unsoundness of mind of an individual accused of crime, *unless their opinions embraced the act originating the accusation, their adjudication would be altogether unjust; for that act might be the hinge on which their estimate of his sanity should turn.* If, on the other hand, they include this act, the onus of proof respecting the guilt or innocence of the party accused is thereby placed in their hands; and we have no grounds for inferring that, under such circumstances, greater unanimity would prevail than is seen in ordinary tribunals. Were they to assume the act as committed, they would thereby identify the question of the accused's sanity with that of his criminality. These, and many other reasons we might adduce, have fully satisfied us that determining guilt or innocence by the voice of the jury, the soundness or unsoundness of mind by the judgment (*i. e.*, according to the evidence) of the physician, is the course best calculated to maintain public confidence and insure public safety."

We believe that the argument so well put by Dr. Williams will commend itself to the judgment of men of all professions, at least of all who do not give way to the irrational impulse to seek for a new and especial intervention of the legislature, in order to obviate every special difficulty that may occasionally arise in practice. We doubt very much, with Dr. Williams, whether the substitution of medical courts, medical *amici curiæ*, or medical juries, would improve the administration of the law in cases complicated with insanity. And of this we are firmly convinced, that no sufficient case has been made out to induce the legislature to entertain so vital a question as that of breaking down the fundamental laws of the country. Let it be borne in mind by those who are ever ready to point a jest or a sarcasm against the ignorance of juries, that ignorance is sometimes less dangerous than imperfect or distempered knowledge. And juries are not always ignorant. Let Englishmen consider whether they had not better trust their liberties to the honest plain sense of twelve of their countrymen than to a medical judge imbued with the abstract crotchets about "parasitical mental growths" and indefinable scales of secondary punishments of Dr. Mayo. But Englishmen are not likely to forget that a jury is the shield interposed between the governing power and the citizen, in order to protect him from arbitrary and illegal assaults upon his liberty. May not a man's liberty be assailed by the false imputation of insanity? *The thing has been done;* it may be done again: and no greater facility for the perpetration of similar outrages could be given than that of withdrawing a man from the cognizance of the jury by setting up the imputation of insanity. But if juries are sometimes "rough instruments of justice," has not the institution borne, during many centuries, a fair comparison with other modes of administering justice? The Ecclesiastical Courts, in which skilled persons preside and adjudicate without the presence of juries, have not worked so well as to have found much favour with the public. Abroad, there is an universal tendency to introduce and extend the jury-system of England.

But why should juries be disqualified on the ground of their ignorance of psychology? *They are not called upon to judge of psychology, but of evidence.* To contend that juries are not competent to deal with questions of insanity, because they are not psychopathic physicians, is to contend that juries are not competent to deal with questions of engineering, because they are not engineers; with questions of law, because they are not lawyers; with questions of science, because they are not scientific; and so on, to the extinction of juries altogether, and the establishment of as many different courts, on the model of the Admiralty or of Ecclesiastical Courts, as there are professions and sciences. Heaven save us from such reforms! We entertain, however, a comforting assurance that the reflecting portion of the public will wait for stronger reasons than have yet been adduced before adopting maxims so revolutionary and so visionary, and that, in the meantime, they will stand by the motto, "Nolumus leges Angliæ mutari."

We have now arrived at the conclusion of our task. We have examined the three great questions raised in the works before us with care, and have expressed our opinions upon them with freedom and with candour. The psychological doctrines of Dr. Mayo are so peculiar in their nature, and so dangerous in the applications proposed, that we have felt it as an imperative duty to expose their fallacy with all the more freedom, because the professional position of their author might obtain for them an extrinsic authority. The psychological doctrines of Dr. Bucknill are more in accordance with the researches of the most trustworthy of modern physicians. The principal objections we have had to urge against Dr. Bucknill's work are not directed against his views of mental pathology. Few physicians of the present day have given more solid contributions to the science of cerebro-mental disease. His labours in this department have been too well directed and too zealously pursued not to have guarded him against gross psychological errors. We have been compelled, however, to criticize, with an appearance of severity, the formidable innovations which he has, upon inadequate grounds, proposed in the administration of the law in cases where the question of insanity is raised. But these medico-legal opinions of Dr. Bucknill bear no necessary relation to his psychological tenets; with the exception of the doctrine of insane responsibility, they are not absolutely incompatible with the soundest principles of medicine. They only betray a mind too eager to contrive artificial reforms, too ready to seek in legislative enactments the remedy for difficulties in practice which arise, not so much from error in the principles of our criminal legislation, as in the differences of opinion about their application in individual cases. This too hasty and meddlesome spirit of change, time and reflection will correct; and we venture to cherish the hope that, one day, Dr. Bucknill will abandon his now favourite doctrines of instituting medical juries; he will admit that it is, on the whole, best that the physician should not travel out of the legitimate paths of medicine, and that he should not seek to be invested with the incompatible attributes of judge, jury, and witness.

Of Dr. Williams's thoughtful, logical, and able essay we have had occasion to speak with almost unqualified praise. His views upon the mooted questions between law and medicine regarding insanity are not less sound and temperate than his fundamental psychological doctrines. Such an essay, so well calculated to spread a just appreciation of the medico-legal relations of insanity throughout the professions of medicine and the law, should not be suffered to remain in its present inaccessible position, scattered over three numbers of a quarterly magazine. We strongly urge upon its author the duty of publishing it in a separate and more convenient form.

